# Optimizing the contributions of nursing and midwifery workforces: #Protect, #Invest, #Together

**DOI:** 10.1186/s12960-021-00577-0

**Published:** 2021-03-02

**Authors:** James Buchan, James Campbell, Carey McCarthy

**Affiliations:** 1 Human Resources for Health/Adjunct Professor UTS, Sydney, Australia; 2 Health Workforce Department, World Health Organization, Geneva, Switzerland

Our journal Call for papers on “Research to support evidence-informed decisions on optimizing the contributions of nursing and midwifery workforces” [1] was announced late in 2019. The aim was to celebrate that 2020 had been designated “The Year of the Nurse and the Midwife” by the World Health Assembly, by setting up a Thematic Series on the two professions. By the time that papers were being submitted to the Call in early 2020, the world had changed. The impact of the Covid-19 pandemic has reinforced the global need for skilled nurses and midwives, and has highlighted their central role in combatting the virus and sustaining access to health systems where possible. In this paper, commemorating the closing of the Call, we reflect on progress made in 2020 in improving policy responses to optimize the contribution of the distinct professions, as well as citing some of the key points emerging from the published papers.

In 2020, for the first time ever, a systematic global survey of the nurse workforce was published. The “State of the world's nursing 2020” (SOWN) [2] was developed by the World Health Organization (WHO) in partnership with the International Council of Nurses (ICN) and the global Nursing Now campaign. Its aim was to provide a “compelling case on the value of the nursing workforce globally” and to set out a forward-looking agenda for adoption and implementation by countries.

The SOWN report drew from data on the nursing workforce in 191 countries. Headline figures were that the global nursing workforce was estimated at 27.9 million, but that it was unevenly distributed across the globe. Over 80% of the world’s nurses were found in countries that account for half of the world’s population. The global shortage of nurses was estimated at 5.9 million nurses in 2018, of which 89% was concentrated in low- and lower middle-income countries.

The report set out a series of recommendations for governments and other stakeholders to address the key nursing workforce challenges, which were summarized in three main areas of action:

1. To invest in the “massive acceleration” of nursing education to address global needs, meet domestic demand, and respond to changing technologies and advancing models of integrated health and social care;
2. To create at least 6 million new nursing jobs by 2030, primarily in low- and middle-income countries, to off-set the projected shortages and redress the inequitable distribution of nurses across the world;
3. To strengthen nurse leadership to ensure that nurses have an influential role in health policy formulation and decision-making, and contribute to the effectiveness of health and social care systems.

The Call for papers for the thematic series in *Human Resources for Health* aimed to inform and support policy makers who were striving to achieve and sustain the maximum contribution from often scarce nursing and midwifery resources. It also aimed to contribute to the growing evidence-base on the roles and impact of nurses and midwives in achieving global development goals. As such, it provided real scope to contribute to improvements in the evidence-base underpinning policy responses, as we highlight later in this paper.

The Call has met its objective: as of January 2021, 20 papers have been published, with several others in the peer review process. The published papers come from all WHO Regions, and cover both professions, and a range of issues; many of the papers make a contribution to one or more of the three main areas of action that were identified in the SOWN report.

The first main area of action identified in SOWN was investment in the “massive acceleration” of nursing education to address global needs, meet domestic demand, and respond to changing technologies and advancing models of integrated health and social care. Papers that have been published under the Call include a focus on policy opportunities in increasing the profile and impact of nursing in Saudi Arabia, with a strong focus on education [3]; a study reporting on the need for alignment and contribution of nursing doctoral programmes to achieve the sustainable development goals in Brazil [4]; an analysis of the experiences of a new cadre of midwives in Bangladesh [5], a scoping review of effective mentoring of working nurses [6], and an assessment of the impact of a mLearning application on nurses’ and midwives’ knowledge and skills in Rwanda [7].

The second main area of action was to create new nursing jobs by 2030, primarily in low- and middle-income countries, to off-set the projected shortages, and to redress the inequitable distribution of nurses across the world. Papers that we have published in the Call include an examination of factors that motivate midwives to remain in their workplace in the Democratic Republic of Congo [8]; a report on evidence-based health workforce planning and unemployed nurses and midwives in Ghana [9]; an analysis of the risks of the precarization (casualization) of the Mexican nursing labour market [10], a study on preferred employment settings of final-year nursing students in Israel [11], and an international review on burnout in nursing, which takes on even greater prominence with the impact of the pandemic [12].

Several papers, notably reviews, provide an evidence-based underpinning for the third area of action: to strengthen nurse leadership to ensure that nurses have an influential role in health policy formulation and decision-making, and contribute to the effectiveness of health and social care systems. These include a scoping review to develop metrics for nursing quality of care for low- and middle-income countries, in order to better demonstrate impact [13], a report on the introduction and extension of nurse prescribing of medicines in 13 European countries, which highlights policy engagement to support advanced practice [14], and a scoping review of the policy implications, positive and negative, of Caribbean nurse migration [15], and a review highlighting the use of advanced practice nurses as a strategy to enable more equitable access to health care in the countries of the Western Pacific Region [16].

As the “Year of the Nurse and Midwife” neared its end, at the resumed 73rd World Health Assembly in November 2021, Member States requested the WHO Secretariat to engage with all WHO Regions, to update the *Global strategic direction for nursing and midwifery* (SDNM), and following consultations, to submit this update to the 74th World Health Assembly in May 2021. WHO has successively developed the SDNM since 2002 for the purpose of delineating policy priorities for the nursing and midwifery workforces, which comprise more than half of most health professional workforces, within the larger human resources for health agenda. The published evidence in this Series, alongside the third edition of the *State of the world’s midwifery* report to be released in May 2021, is therefore of immediate value to inform the identification and prioritization of global strategic actions to optimize the nursing and midwifery workforces across differing national contexts.

Immediately following the decision of the 73rd WHA, a ‘draft for consultation’ of the SDNM 2021–2025 was disseminated globally [17]. A series of Regional and global consultations with government and all relevant stakeholders commenced in December 2020 and will be followed by review by WHO Member States in February 2021. This is the first occasion for a draft SDNM to be subject to the Member State governance process: prior versions have been produced by expert advisory groups and WHO, and published as technical guidance documents.

The draft SDNM 2021–2025 comprises the three major areas for investment—education, jobs, and leadership—and includes a crucial fourth area: the practice of nurses and midwives in their work environments. Under each main area, a forward-looking “strategic direction” is articulated along with two to four prioritized policy actions. In total, these provide 11 evidence-based policy priorities for all countries to consider. For instance, what measures can be taken to provide jobs for unemployed nurses or what actions will ensure job creation and retention in rural health settings? The monitoring framework allows for ministries of health and government chief nursing and midwifery officers within them, to use pre-existing reporting mechanisms to continuously assess progress.

As noted above, the SDNM will be submitted to the 74th World Health Assembly for its consideration in May 2021. The last substantive agenda item on nursing and midwifery was 10 years ago in May 2011: WHA 64/7 Strengthening Nursing and Midwifery [18]. It is therefore an opportune time to leverage the evidence-base, the global policy debate and the public recognition of the value of nurses and midwives in protecting the world’s health.

Governments have signalled their appreciation for nurses and midwives in designating 2020 as WHO’s Year of the Nurse and the Midwife. The evidence-base, supported by this Thematic Series, is improved. The continuing COVID-19 global pandemic has carried us into a new phase of appreciation and respect for nurses, midwives and all health and care workers at the forefront of local, sub-national and national responses; resulting in a decision by the 73rd World Health Assembly to designate 2021 as the International Year of Health and Care Workers. At the heart of this second Year is a call for action that if we aim to manage the pandemic, maintain health services, improve health workforce readiness, education and learning, and roll-out COVID-19 vaccination equitably, the world must protect and invest in all health and care workers.

As country governments, treasury officials and International Financing Institutions consider and embark upon decisions on where and how to invest billions of dollars in social protection and economic recovery in response to the COVID-19 pandemic, we urge all relevant stakeholders to heed this call for the world’s nurses, midwives, health and care workers and to play their part: #Protect, #Invest, #Together.

## Data Availability

Data sharing is not applicable to this article as no datasets were generated or analysed.
