# Correction to: Optimizing the contributions of nursing and midwifery workforces: #Protect, #Invest, #Together

**DOI:** 10.1186/s12960-021-00583-2

**Published:** 2021-03-22

**Authors:** James Buchan, James Campbell, Carey McCarthy

**Affiliations:** 1 Human Resources for Health/Adjunct Professor UTS, Sydney, Australia; 2 Health Workforce Department, World Health Organization, Geneva, Switzerland

## Correction to: Hum Resour Health (2021) 19:26 https://doi.org/10.1186/s12960-021-00577-0

Following the publication of the original article [1], the authors would like to correct the number of papers in the following sentence. The change has been highlighted in **bold typeface**.

The sentence currently reads:

The Call has met its objective: as of January 2021, 2020 papers have been published, with several others in the peer review process.

The sentence should read:

The Call has met its objective: as of January 2021, **20** papers have been published, with several others in the peer review process.

The original article [1] has been corrected.
